# A tale of four countries: How Bowlby used his trip through Europe to write the WHO report and spread his ideas

**DOI:** 10.1002/jhbs.22016

**Published:** 2019-11-19

**Authors:** Frank C. P. van der Horst, Karin Zetterqvist Nelson, Lenny van Rosmalen, René van der Veer

**Affiliations:** ^1^ Department of Psychology, Education & Child Studies Erasmus School of Social and Behavioral Sciences, Erasmus University Rotterdam Rotterdam The Netherlands; ^2^ Child Studies, Department of Thematic Studies Linköping University Linköping Sweden; ^3^ Centre for Child and Family Studies Institute of Education and Child Studies, Leiden University Leiden The Netherlands; ^4^ Department of Psychology University of Magallanes Punta Arenas Chile

**Keywords:** attachment theory, Bowlby, maternal deprivation, mental hygiene, separation, WHO

## Abstract

Attachment theory, developed by child psychiatrist John Bowlby, is considered a major theory in developmental psychology. Attachment theory can be seen as resulting from Bowlby's personal experiences, his psychoanalytic education, his subsequent study of ethology, and societal developments during the 1930s and 1940s. One of those developments was the outbreak of World War II and its effects on children's psychological wellbeing. In 1950, Bowlby was appointed WHO consultant to study the needs of children who were orphaned or separated from their families for other reasons and needed care in foster homes or institutions. The resulting report is generally considered a landmark publication in psychology, although it subsequently met with methodological criticism. In this paper, by reconstructing Bowlby's visit to several European countries, on the basis of notebooks and letters, the authors shed light on the background of this report and the way Bowlby used or neglected the findings he gathered.

## 1 INTRODUCTION

After World War II there was widespread concern over the psychological consequences of the war on children's emotional development (Shields & Bryan, 2002; Zahra, 2015). The results of more than 5 years of fighting, bombing, starvation, separation, and loss were felt throughout the continent. During the war children in German‐occupied territory were deported, orphaned, or went into hiding—with or without their parents. In Britain, children were evacuated without their parents from war‐stricken areas to protect them against air raids (Downs, 2006; Wick, 1988; Wick, 1990; Zetterqvist Nelson, 2016), see also (Bowlby, Miller, & Winnicott, 1939). For those who could stay with their parents, there were still other concerns: hunger, cold, and fear were omnipresent. In total, the number of orphans in Europe at the end of the war was estimated at 13 million (Macardle, 1949).

In contrast to ideas at the end of World War I, when the focus had been on addressing children's physical problems like undernourishment and disease, psychologists and psychiatrists now worried about the consequences for children's social and emotional development. It was generally believed there were potentially long‐lasting effects that needed to be addressed. The idea that inadequate emotional adjustment during infancy and childhood could affect mental health was brought forward by the Mental Hygiene Movement, which originated in 1909 in the US and expanded to Canada in 1918. Partly influenced by psychoanalytic thinking, Mental Hygiene was essentially an approach that tried to prevent mental illness or maladjustment in adulthood by promoting healthy socialization during childhood. The movement flourished during the Interbellum, with representatives from more than 50 countries, many European, present at the first International Congress in Washington D.C. in 1930 (Richardson, 1989).

When diplomats from all over the world met in 1945 to form the United Nations, partly in view of the new ideas on global mental health issues, such as derived from the Mental Hygiene Movement and psychoanalytic views, they already discussed setting up a global health organization. WHO's Constitution (World Health Organization, 1946) was adopted by the International Health Conference held in New York from 19 June to 22 July 1946 and came into force on April 7, 1948. It stated that “health is a state of complete physical, mental and social well‐being and not merely the absence of disease or infirmity” and that “the enjoyment of the highest attainable standard of health is one of the fundamental rights of every human being without distinction of race, religion, political belief, economic or social condition” (p. 1). Against the backdrop of two World Wars, it was affirmed that “the health of all peoples is fundamental to the attainment of peace and security” (World Health Organization, 1946).

From its conception, the WHO was a leading international organization that functioned as a knowledge broker and influenced the global scientific agenda in mental health care (cf. Turchetti, Herran, & Boudia, 2012). For children, healthy development was seen to be of fundamental importance, inspired by the idea that society as a whole would profit and recurrence of profound misery as seen in the first half of the twentieth century could be avoided by this new, “healthy” generation. No wonder, then, that the WHO wished to issue a report on the care of homeless children. Its author would be the London based British child psychiatrist and psychoanalyst John Bowlby (1907–1990), who subsequently became known as the founder of attachment theory.

The problem of homeless and familyless children was given much attention in psychoanalytic circles in Great Britain at the time (cf. Van der Horst & Van der Veer, 2009a; 2009b; Van der Horst & Van der Veer, 2010), in particular by Dorothy Burlingham and Anna Freud, who worked with very young children in the Hamstead War Nurseries in London during World War II. In their studies, (Burlingham & Freud, 1942; Burlingham & Freud, 1944) posited that it was of the utmost importance for the child's personality formation to develop attachments with adults. Although Bowlby and Anna Freud were no allies and were critical of each other's work, Bowlby was well aware of Anna Freud's work with regard to problems of institutional care that revolved around issues of lack of family relationships (Robertson & Bowlby, 1952).

To secure the most recent information on the topic, Bowlby first traveled from England to the European continent to meet with leading experts in the field of child development. However, Bowlby was no novice in the field: Since the early 1930s he had held strong opinions about the origin of mental health problems in children, which in his view could often be attributed to an inadequate relationship with the child's mother (Van der Horst & Van der Veer, 2010). In Bowlby (1939), for example, he had argued that “the emotional bond between child and mother is the basis for all further social development” (p. 3) and that prolonged physical separations from the mother cause children to feel “deserted and betrayed.” As a result, they would develop “a lack of trust and disregard for other people” (p. 5). This was a position that Bowlby, in his own words, did not subsequently change “in any material way” (Bowlby, 1958, p. 248) over the years. It comes as no surprise, therefore, that in his WHO report Bowlby reached the conclusion that “what is believed to be essential for mental health is that the infant and young child should experience a warm, intimate, and continuous relationship with his mother (or permanent mother‐substitute) in which both find satisfaction and enjoyment” (Bowlby, 1952, p. 11). What is perhaps more surprising is that a report about the problems of homeless children ended in this conclusion.

Issuing a report about homeless children provided Bowlby with an ideal opportunity to spread and discuss his ideas internationally and to gather evidence that confirmed his view. Bowlby himself considered the European research trip and the subsequent trip to the USA as formative: “[T]hose 5 months I worked for [the] World Health [Organization] were very crucial ones for me, because first of all, I got to know a lot of people, secondly, I had a chance to read up the literature which I had not been able to do before and thirdly, to put my ideas together on paper” (Senn, 1977, p. 18).

Bowlby's eventual WHO report on maternal care and mental health was widely distributed and discussed. The report became an immediate international success, receiving many favorable reviews^1^ and was translated into 12 languages (Bowlby, 1986). The abridged paperback version *Child care and the growth of love* (Bowlby, 1953) sold nearly half a million copies (Holmes, 1993; Karen, 1994). However, Bowlby's basic assumption that separation from the mother was the origin of all of the homeless children's problems was not accepted in some countries (e.g., Sweden; see Zetterqvist Nelson, Van der Horst, & Van der Veer, 2017) and subsequently met with increasing criticism from researchers within the field (Casler, 1961; Clinton, 1986; Mead, 1962; Rutter, 1972; Wootton, 1959), researchers drawing on feminist theory (Morgan, 1975) and, more recently, historians of science (Vicedo, 2013). Moreover, 10 years later the WHO issued another report, called *Deprivation of maternal care: A reassessment of its effects*, which pointed out the limitations of Bowlby's research and was critical of his conclusions (Ainsworth et al., 1962).

In this article, we aim to shed light on how the WHO report came into being and the specific circumstances under which Bowlby wrote the report. We explore in detail how Bowlby, as a “traveler in knowledge” (cf. Harris et al., 2015), used the WHO to further develop and spread his ideas on maternal deprivation and the significance of early mother–child bonding. In our examination, we follow in Bowlby's footsteps, traveling through post‐war Europe, starting at the WHO headquarters in Geneva, then visiting France, The Netherlands, and Sweden, before going back to Switzerland. Based on archival material—consisting of Bowlby's notebooks and personal letters, produced during his 6 weeks of traveling—and on the final report, we have mapped out in detail the visits to each country, including the dozens of meetings with clinicians, researchers, and policy‐makers. As we shall see, Bowlby used the WHO as a platform to communicate and disseminate his ideas about early mother–child bonding and largely disregarded evidence that was at variance with his views or that he found irrelevant. But first, we shall briefly sketch the outlines of attachment theory as developed by Bowlby and Ainsworth.

### 1.1 Bowlby and attachment theory

The outlines of Bowlby's attachment theory were originally formulated in the 1950s and further developed together with the American‐Canadian psychologist Mary Ainsworth (1913–1999) in the 1960s (Van Rosmalen, Van der Horst, & Van der Veer, 2016). But, as said before, many of Bowlby's ideas were shaped before the war: in part by his distant upbringing (Van Dijken, 1998) and by his professional work as a clinician in child guidance, where he noticed that mental problems and delinquency may be rooted in an inadequate emotional relationship with the principal caregiver (Van der Horst & Van der Veer, 2010). During the Interbellum, a period of extremism and international aggression, Bowlby also worked with Labor politician Evan Durbin (1906–1948) on the psychological causes of war (Durbin & Bowlby, 1939; cf. Mayhew, 2006; Thomson, 2013). From these formative experiences, Bowlby formulated attachment theory, now considered one of the major theories in developmental psychology and developmental psychopathology.

Attachment theory, in which Bowlby tried to explain how and why children form bonds with their parents and caregivers, holds that for healthy emotional development into adulthood, children need a consistent nurturing relationship with a caregiver, who is sensitive to the child's emotional needs. If such a caring relationship is missing, or a caregiver is unreceptive to the child's signals, it may result in abnormal child behavior and may even lead to psychopathology. Bowlby based his early ideas on the importance of early “object relations” (i.e., relations with caregivers) on retrospective evidence, which he collected while working with juvenile delinquents at the London Child Guidance Clinic. In *Forty‐four Juvenile Thieves*, Bowlby (1944) tried to corroborate his view that a “broken mother‐child relationship” is the source of later delinquency and mental disturbance. In close collaboration with British ethologist Robert Hinde, Bowlby found theoretical underpinning for his ideas in the work of leading European ethologists Konrad Lorenz and Niko Tinbergen, which laid the foundations of Bowlby's conceptualization of a biological and evolutionary basis for the mother–child relationship (Van der Horst, Van der Veer, & Van IJzendoorn, 2007). Bowlby made the move from theoretical and observational claims to experimental evidence through his interactions with American animal psychologist Harry Harlow, with whom he was in contact from 1957 through the mid‐1970s. Bowlby profited highly from Harlow's experimental work on the effects of separation in infant rhesus monkeys (Van der Horst, LeRoy, & Van der Veer, 2008). This eventually resulted in the formulation of attachment theory in his trilogy *Attachment, separation, and loss* (Bowlby, 1969; Bowlby, 1973; Bowlby, 1980).

### 1.2 Bowlby's appointment as a consultant for the WHO

To be appointed as a consultant for the WHO was prestigious and it is unlikely that Bowlby, who was fairly unknown at that stage of his career, would have been selected if it hadn't been for his good friend Ronald Hargreaves (1908–1962). Hargreaves and Bowlby had met during the war when both were army psychiatrists, and after the war worked together at the Tavistock Clinic. In 1949, Hargreaves was appointed Chief of the Mental Health Section at the WHO in Geneva (Rees, 1963). It seems that Hargreaves was impressed by Bowlby's (1944) study of young thieves, whose behavior Bowlby believed to be caused by separations from the mother, and asked him to do a study on juvenile delinquency for the WHO. Bowlby refused (Van Dijken, 1998) and the study (Bovet, 1951) was eventually conducted by the Swiss child psychiatrist Lucien Bovet (1907‐(1951). Bowlby later said that he told Hargreaves that the study on delinquency did not “suit [him] too well, but if anything else to do with children [would] turn up, [he] might find it of interest” (Senn, 1977). That specific opportunity came within a month or two, as the Social Commission of the United Nations decided to carry out a study of the needs of homeless children in 1948. Hargreaves was eager to get the WHO involved in reporting on the psychiatric aspects of this issue and again turned to Bowlby (Karen, 1994; Senn, 1977; Van Dijken, 1998). This time Bowlby accepted and during the entire enterprise, he would work closely together with Hargreaves. It was decided that Bowlby would travel to the USA and various European countries to discuss the problems of homeless children with the experts in the field, to see how the local authorities and experts dealt with the problems and, if possible, to gather research findings.

For the preparation of his trip, Bowlby could rely on several of his colleagues and he believed that the preparations for his visit had been very adequate: “As a result of all the good advice I received from [Noel Hunnybun] and Sybil [Clement Brown], J[ack] R[ees], Ronald [Hargreaves] and Elliott [Jaques], I seem to have made as good a plan as is possible… and to have arranged to see all the right people. Indeed everyone is slightly amazed that I am so well informed of what is going on and who is who.”

In January 1950 Bowlby took up his temporary appointment with the World Health Organization and during the late winter and early spring visited several countries in Europe—France, the Netherlands, Sweden, Switzerland, and the United Kingdom—and the United States of America. In discussions with the people he met, he “found a very high degree of agreement existing both in regard to the principles underlying the mental health of children and the practices by which it may be safeguarded. In compiling th[e] report [his] task has thus been to do justice to an extensive literature and to bring out the many points of importance to which [his] attention ha[d] been drawn; little time ha[d] had to be expended in reconciling divergent views” (Bowlby, 1952, p. 6).

This was a slightly optimistic conclusion, as we will see in the chronological reconstruction of Bowlby's trip through Europe. It all started with Bowlby's departure from Northolt Airport^2^ in London to Geneva, Switzerland, to the WHO headquarters, where he would meet with Hargreaves to work out the details of his program.

## 2 BOWLBY'S EUROPEAN RESEARCH TRIP: A CHRONOLOGY^3^

### 2.1 Prelude: preparations in geneva

After landing in Geneva on January 9, 1950, and spending the afternoon on official form filling, Bowlby first had a talk with Ronald Hargreaves and then was introduced to several people working at the WHO. Apparently, his program for the weeks to come was not what he expected. Bowlby was to travel through Europe for several weeks first, before going to the USA As he wrote to his wife Ursula, this was “inconvenient as [he] had no clothes for 6 weeks on the Continent, no[r] any Recordon^4^ Clearly, Bowlby had expected to fly back to England and go to the USA, before he would visit any of the European countries. Bowlby commented that “it was silly of [Hargreaves] not to have let him know earlier.” As a result, in the days following his arrival, Bowlby wrote to Ursula to get additional clothes and made arrangements with his colleagues to get his Recordon flown in.

On his first evening in Geneva, Bowlby took up residence in a modest fifth‐floor apartment in Hotel Eden, which was conveniently located as it was only about a 10‐min walk from the WHO. In the *Palais des Nations*, the WHO headquarters, Bowlby had his own desk and a research assistant. This research assistant was a young Swiss psychologist named Philippe Kocher, whose task was to find and abstract literature on juvenile delinquency for Bovet's study and on homeless children for Bowlby's study (Bowlby, 1952; Van Dijken, 1998). Bowlby's impression of Kocher was that of “a very nice and bright young man whose attitude to the problems of psychiatry is not unlike [my] own.” Kocher's only shortcoming according to Bowlby was that he was a communist, although “not an aggressive one” who might “get cured 1 day!”^5^ Bowlby himself supported the Labor Party, a preference he had held at least since the 1930s, when he was befriended with the Labor politician Durbin (Van der Horst et al., 2007).

During the first days in Geneva, Bowlby spent time at the WHO headquarters “rather hectically planning visits and whom to see, structuring the content of the report and generally acclimatizing myself to this fabulous organization.” From Bowlby's correspondence, it becomes clear that he had daily discussions with Hargreaves about the probable shape of the report and that they found themselves in close agreement. In all, he felt the scope of his work seemed to fall under three main heads. First of all, he was interested in the causes of homelessness. Secondly, he wanted to get scientific evidence regarding the effects of homelessness on children's personality and cognitive functioning. And finally, he was looking for psychological knowledge regarding the therapeutic handling of homeless children, which would cover all problems connected with observation centers, adoption procedures, foster homes, and institutions. Bowlby's idea was “that whilst scores of people [had] strong opinions about all these things, relatively few [had] well‐validated evidence in favor of their opinions. It [might] well be that the principal conclusion of the report [would] be that a big gap lies between opinion and evidence, which need[ed] filling by research.” Characteristically, Bowlby was not even sure that his conversations with fellow researchers would yield many new insights. He was actually worried “about the danger of racketing about seeing too many people—half of whom [would] be pretty stupid” and added that “in some ways I regret having to spend so much time visiting various capitals, since [it is] very doubtful whether I shall pick up much of value which I do not know about already, though this may prove unduly cynical.” Bowlby realized, however, that Hargreaves had excellent contacts in the field and that he would “use his not inconsiderable influence to get people to support [me]”, which would allow him to continue or even expand his research program.

In Geneva, the first opportunity for research funding came on January 12, when Bowlby had lunch with Lady Allen of Hurtwood (1897–1976). Lady Allen was a strong advocate for child welfare and had been a driving force behind the passing of the Children Act in England in 1948. This Act established childcare services and made local authorities responsible for the care of children who had lost their parents or whose parents for whatever reason could not care for them. The Act stipulated that this should always be done in close collaboration with the families themselves. In fact, Bowlby's own Children's Department at the Tavistock was an indirect result of this very Act and the focus on families was certainly in line with his views. Furthermore, Lady Allen was associated with the United Nations International Children's Emergency Fund (UNICEF), which increased Bowlby's hope to get grants for his separation research at the Tavistock Clinic. The first meeting with Lady Allen was followed by another lunch meeting a week later in Paris, on January 18, and it proved to be time well spent, because eventually Bowlby would receive funding for his research on separation (see below).

Above we called Hargreaves a friend and, indeed, it seems that Hargreaves was more than just a colleague and sympathizer. For example, in his notes, Bowlby mentioned that he spent a very pleasant evening with Ronald and Eva Hargreaves in their home, which he described as a small chateau in a neighboring village on the French side of the border. From the notes, it also becomes clear that the families, both parents, and children, knew each other well. On the last day of his stay in Geneva, before he left for Paris, Bowlby again joined the Hargreaves family and “went up the Jura hills above the fog into the most brilliant sunshine and a little snow, with… lovely views from the Alps for winter sports” as there was “enough snow to do a little skiing and some tobogganing.” As is often the case, scientific developments depend in part on personal relationships.

### 2.2 Paris: “psychoanalysis all the way”

On January 16, Bowlby arrived in Paris, where he resided in Hôtel Belfast on Avenue Carnot, an older middle class hotel near the Arc de Triomphe. Again his hotel was located close to non‐profit and governmental organizations, such as UNESCO and the ministries. Bowlby, in a letter to Ursula, admitted that he was “in rather down spirits.” Somehow, he suspected that the next 3 weeks would be the worst of the whole tour and he again expressed his fears that he would not learn very much and resented the prospect of having “to be polite and pretend what they t[old] [him] [wa]s ever so important.”

Bowlby's first day in Paris confirmed his suspicions: Letters to various people written in Geneva had not arrived and no one seemed to be accessible by phone. Fortunately, someone at UNESCO provided him with a report on the education of war‐damaged children, which was to be published the week after. Bowlby spent most of the morning reading it in the UNESCO library and found that it did not contradict his own views, which was of some importance given that it was a report in the same series as his own report to be.

The report to which Bowlby was referring was a study on war‐handicapped children by Thérèse Brosse (1902–1991), head of the Education Department of UNESCO. In her study, Brosse (1950b) reported on the educational needs of war‐handicapped children, which included not only physical needs but aspects of emotional and mental development as well. According to Brosse, “education is… conditioned by the cultural environment and the different educators (the family, the school, various other groups)—a social structure which not only affects the teaching the child receives but also governs the harmonious development of his body, his emotions and his intelligence” (p. 14). Brosse emphasized the importance of “social situations to which many abnormalities in children are attributable” (p. 142), a view that certainly was in line with Bowlby's thinking at that time. In another study for UNESCO, on homeless children, Brosse (1950a) also pronounced views similar to Bowlby's, as she stated that “while the reports tell of disturbances in character resulting from war, they show also the fundamental part played in their causation by rupture of the family tie.” It is unclear whether Bowlby actually met with Brosse in person, as his preliminary schedule suggested. An entrance in one of Bowlby's notebooks shows a question for a “Discussion [with] Dr Brosse” about “what research [was] going on,” but Bowlby made no further notes and he did not mention talking to her anywhere in his letters. Bowlby did, however, mention the work of Brosse in his WHO report, specifically her claim that rupture of the “family tie” played a fundamental part in disturbances of a child's character (Bowlby, 1952, p. 44).

In the next few days, Bowlby met with several leading psychoanalysts and acquainted himself with the state of child guidance work in Paris. He visited the clinic of the professor of child psychiatry Georges Heuyer (1884–1977), a clinic that he described as “a frightful shambles of a place in an old hospital.” Here he was shown around by Heuyer's assistant Serge Lebovici (1915–2000), “an energetic, young analyst” and an enthusiast for Melanie Klein's work. According to Bowlby, Lebovici worked along his lines, “although the conditions make it almost impossible.” Lebovici would later become a prominent figure in the French psychoanalytic movement, mainly interested in child psychoanalysis, where he introduced the ideas of Klein and Winnicott, and would become an advocate for Bowlby's ideas on attachment, who made a great impression on him during Bowlby's short visit to Paris (Geissmann & Geissmann, 2005). Like Robertson and Bowlby (Van der Horst & Van der Veer, 2009a; 2009b), Lebovici researched the problems children meet in hospitals (Lelong & Lebovici, 1955) and in 1962 he still by and large defended Bowlby's WHO approach and findings, although he made the sobering remark that it would “be highly dangerous to attribute the overwhelming majority of emotional and mental disorders in adolescents and adults to such deprivation… Psychopathological structures that have been built up slowly and constantly reshaped obviously cannot be due to a single event, however serious its significance, even if it occurred at a decisive time in the establishment of object relationships” (Ainsworth et al., 1962, pp. 90‐91).

Bowlby also met with Daniel Lagache (1903–1972), a professor of psychology at the Sorbonne University and another leading psychoanalyst, who, together with Jacques Lacan (1901–1981), sought to renew psychoanalysis. Apparently, Lagache was “a great enthusiast” for Bowlby's work as he organized a dinner at his house on Boulevard Saint‐Germain in Bowlby's honor. In his table speech, Lagache mentioned that he was arranging a French translation of Bowlby's (1944) article about juvenile thieves to appear in *Revue française de psychanalyse*, the French journal of psychoanalysis.

On the basis of these encounters, Bowlby concluded that French “child guidance exists merely in little patches, odd clinics springing up in various parts of Paris, mostly in hospitals.” What surprised him most was that French child guidance operated on a thoroughly psychoanalytic footing. In itself, this betrayed Bowlby's ignorance about French psychiatry: France was and still is one of the few countries in the world where the influence of orthodox or Lacanian psychoanalysis is exceptionally strong both in theory and practice (Botbol & Gourbil, 2018). Although Bowlby by this time had seen everyone he had intended to see, the most fruitful meetings were yet to come: those with Jenny Roudinesco (1903–1987) and her assistant Geneviève Appell (b. 1924).

On January 19, Bowlby had dinner with Jenny and her husband Alexandre Roudinesco in their apartment in a fashionable part of Paris, which was “heavy‐thick with [paintings by] Dufy, Van Dongen^6^ and others.” Bowlby's first impression of Jenny Roudinesco was that of “a fashionable lady of about 45” who reminded him a good deal of his former training analyst Joan Riviere (Van Dijken, 1998). As it turned out, Jenny Roudinesco's work greatly appealed to him. In his report to Noel Hunnybun, Bowlby mentioned that “[t]he most interesting work going on [here] is that by Madame Roudinesco, a child psychiatrist who is also training to be an analyst. She and her psychologist [Geneviève Appell] are doing some systematic work in a residential nursery which is attached to the hospital in which [Roudinesco] works.” The residential nursery mentioned here was called *La fondation Parent de Rosan*, a nursery where children between 0 and 3 years of age were in care and that was linked to the *Ambroise‐Paré* hospital, where Roudinesco had been in charge of the pediatric unit since 1946. Several days after their first encounter, Bowlby visited the residential nursery himself and reported that “[t]hey get… mostly neglected children from the streets who have to be placed and many of them are in a very bad way… They are using Gesell's tests for development and have got some quite interesting results. They are familiar with the work of [René] Spitz and are to some extent repeating it.” Impressed by the work done in the clinic, Bowlby immediately expressed the hope that Geneviève Appell would be able to visit the Tavistock Clinic that same year. This would be the start of very fruitful cooperation between Bowlby's team and the French team led by Jenny Roudinesco (Dugravier & Guedeney, 2006).

Just a couple of months after their first meeting in Paris, Roudinesco, Appell, and Bowlby successfully applied for a grant from the *Center International de l'Enfance*. Their success again owed much to Hargreaves who was able to get people to support Bowlby and his team. In the following years, the two teams would be able to study “the repercussions of separations from the mother among young children” (Roudinesco, David, & Nicolas, 1952, p. 66). The actual implementation of the project became the joint responsibility of pediatrician and psychiatrist Myriam David (1917–2004) and Geneviève Appell, who would on a regular basis across the Channel to meet with Bowlby and his team at the Tavistock Clinic. Appell would also attend all four of the interdisciplinary Tavistock meetings organized by Bowlby (Van der Horst, 2011) and kept in touch with Bowlby until his death in 1990 (G. Appell, personal communication, April 22, 2016). In the WHO report, (Bowlby, 1952, pp. 19‐21) mentioned the work of Roudinesco and Appell on the IQ development of children growing up in family settings vs. institutional settings and found proof for the adverse effects of deprivation in institutions.

Although Bowlby's schedule was stuffed with appointments, he did find some time for relaxation in Paris. He visited the Louvre, which since his last visit in 1933 had “been rehung extremely well and there [was] a wealth of good pictures, especially Italian.” But the most remarkable visit was a night at the famous cabaret music hall *Folies Bergère*, where Bowlby and two of his WHO colleagues saw Josephine Baker perform in a show that combined both comedy and eroticism.

All in all, however, Bowlby was not greatly impressed by the clinical work he had seen in Paris. The work of French psychoanalysts such as Heuyer and Lagache did not appeal to him and was not even mentioned in the final WHO report. As said before, Bowlby probably hadn't expected the influence of orthodox psychoanalysis, with its reservations about empirical studies, to be that strong in France. But it was and it would later take Elisabeth Roudinesco, daughter of the Roudinesco couple, two volumes to describe the history of psychoanalysis in France (Roudinesco, 1982; Roudinesco, 1986). On the other hand, Bowlby was delighted by his encounters with Jenny Roudinesco and the prospects of joint research. This “French connection” was no doubt one of the great achievements of Bowlby's European research trip. Bowlby was now bound for The Netherlands, a visit that he expected to proceed with fewer language difficulties if only because “no one will expect me to speak Dutch!”

### 2.3 The Netherlands: “Child guidance flourishes, but no research”

On January 22 Bowlby arrived in Amsterdam. The next day he transferred to The Hague, where he stayed at the Hotel Terminus on the Stationsweg. Bowlby admitted that “it [wa]s really rather silly staying in The Hague, but as… government officials live here it is difficult not to.” The problem was that most experts lived elsewhere in the Netherlands, which forced Bowlby to spend two or more hours on the train each day. One of the government officials he met was Cornelis van den Berg (1892–1957), director‐general for International Health Affairs and, as a member of the Executive Board of the WHO, a liaison between WHO, Bowlby, and Dutch authorities. In previous weeks, Bowlby had met with Van den Berg several times, in both Geneva and Paris.

On his first full working day in The Netherlands, January 23, Bowlby visited the Ministry of Social Affairs, where he was kindly received. In the afternoon, he spoke with psychiatrist Arend van Meurs (1914–1997) for an hour and a half, director of the Child Guidance Clinic in The Hague. Bowlby's impression was that they were doing good work. In the evening, he had dinner with psychiatrist Johannes van der Spek (1886–1982). Van der Spek was the director of *Maasoord*, a psychiatric hospital in Rotterdam, and a member of the Central Commission of Public Health, an advisory body on health issues for the Dutch government. During dinner it became clear that Van der Spek had organized “a proper program” for Bowlby's stay, so the “day was not wholly wasted.”

Bowlby spent the next day, January 24, seeing the Amsterdam Child Guidance Service, which was run by psychoanalyst Theo Hart de Ruyter (1907–2001). According to Bowlby, the “Amsterdam C[hild] G[uidance] service [would] put London to great shame; the Health Service is on an infinitely better scale than we have seen in the London area.” He also had an excellent talk with “Tavvy oriented” Psychiatric Social Worker Eus Lekkerkerker (1899–1985) who, between 1924 and 1926, studied women's reformatories in the United States (Lekkerkerker, 1931) and during her stay became hugely inspired by the Mental Hygiene Movement. Upon her return, she sought funding for training in the USA of a psychiatrist and a psychiatric social worker and for the implementation of these new ideas on prevention in mental health care in The Netherlands. As a result, psychiatrist Nel Tibout (1899–1968) was elected to spend parts of 1927 and 1928 in the USA to do research on Child Guidance work. On the basis of these experiences, Lekkerkerker and Tibout founded the first Dutch Child Guidance Clinic in Amsterdam in 1928 with Tibout as director (Van der Horst, 2014). Based on Mental Hygiene principles, with a clear grounding in psychoanalysis, the clinic provided preventive care to children with problems varying from bed‐wetting to behavioral problems and from chronic frowardness to sibling rivalry. The clinic also reported on families involved in court cases on child custody, provided school counseling and supervised two special day schools for neurotic and delinquent children.

After visiting the CGC, Bowlby had a meeting with Arie Querido (1901–1983), social psychiatrist and responsible for public mental healthcare in the municipality of Amsterdam. Bowlby clearly saw the benefits of having a psychiatrist as head of Community Health Services, as he felt the city was fairly psychiatrically minded and offered good support. Querido's intention was to increase the psychiatric services in Amsterdam, but a shortage of people was holding back expansion. To this purpose, Querido was trying to get people trained as psychiatric social workers, by Tibout, for example. With regard to the quality of work done in Amsterdam, Bowlby commented that people were “obviously working on comparable lines to ours and have similar conceptions. They are enthusiastic about keeping children in their homes, helping the parents and helping schools to deal with them.” Indeed, Bowlby's (1952) view that “children thrive better in bad homes than in good institutions” (p. 68) was certainly in line with that of (Querido, 1933; cf. Heerma van Voss, 1991), who had done research on “antisocial families,” placed in *Zeeburgerdorp*, a separate and distant district of Amsterdam. In his study, Querido stated that the causes of family failure were not due to genetic deficiencies but to the social characteristics of these families and the ineducable psychopathic character of the parents. In the WHO report, Bowlby mentioned Querido's division of problem families in three groups: (a) those which, provided economic and medical help can be given, can become once again effective social units; (b) those which may require some degree of permanent help but which can respond favorably to it; (c) those which all ordinary social measures are powerless to assist. Bowlby fully agreed with Querido that workers need psychiatric insight to identify psychopathology in parents and he concluded that there was no solution to the problem yet but that Querido's proposal for placing whole families under supervision and restraint was “most realistic and constructive.” Yet, a program of this kind would in almost all countries require legislation, for it involved “a serious infringement of personal liberty and offers possibilities of abuse” (Bowlby, 1952, p. 90). In The Netherlands, such legislation was being drafted at that time.

The next day, Bowlby spent some time in Miss Lekkerkerker's office on the Prinsengracht dictating letters, whereas enjoying the view over the frozen canals. In the evening he had dinner with Tibout, who appears to have been a good friend, as Bowlby, whereas in Paris, specifically asked his wife to send photos of their children to show Tibout. Bowlby and Tibout had met several times before at international conferences in Zürich, in Amsterdam, where Bowlby read a paper on his study on juvenile delinquency (Bowlby, 1944), and in London, where Tibout was part of a symposium on “Aggression in relation to normal and pathological emotional development” together with Anna Freud and child psychiatrist Fred Allen (1890–1964). Naturally, Bowlby, through his membership of the *British Psychoanalytical Society*, was well acquainted with the work and ideas of Anna Freud and Fred Allen would actually be Bowlby's host several weeks later during a short stay in Philadelphia in March 1950. It is clear that Tibout and Bowlby held quite similar views: both advocated—in spite of the prevailing psychoanalytic *Zeitgeist*—an eclectic approach to child psychiatry, both sought cooperation with other disciplines, and both highly valued direct observation of child behavior (Simpelaar, 2012; Van der Horst, 2014). Dinner was very agreeable and after some “gossip” and exchange of personal matters, Bowlby had to hurry to catch his train back to The Hague.

On January 26, Bowlby went to Amersfoort to visit the children's institution of *Zandbergen*, which was “run by an ex‐inmate.” *Zandbergen* was an institution that took up children in a therapeutic society that—as much as possible—resembled the family environment (see below for a similar institute at Skå in Sweden). The institution prepared children for placement with foster parents. Its director was Daan Mulock Houwer (1903–1985), who had been placed in *Zandbergen* as an orphan at the age of 14 and later became first a social worker in *Zandbergen* and then director of the institution. On the basis of his lengthy discussion with Mulock Houwer, Bowlby wrote in his notebook that satisfactory placement of foster children was possible under the following circumstances: “diff[erent] sex to other child of same age; if same‐sex optimal age diff[erence] is 4 y[ea]rs ±; if fantasies of a dead child [etc.] are adequately discussed; parents must be young enough for the child; simple friendly household.” In the WHO report, (Bowlby, 1952, p. 127) mentioned Mulock Houwer's preliminary findings on optimal age and sex differences for foster placements as established facts.

In the evening of January 26, Bowlby traveled back to Amsterdam to have dinner with Tibout and Dientje de Leeuw‐Aalbers (1908–1957) who told him about the Jewish children who were saved from the Nazis but lost their parents. On the basis of what Tibout and De Leeuw told him, Bowlby concluded that “children made tremendously strong att[achments] with foster m[others].” It is interesting to note that Bowlby used the term “attachment” for the bond between mother and child as early as 1950, years before the formulation of attachment theory (cf. Van Rosmalen et al., 2016).

The next day, January 27, Bowlby visited the psychiatric–neurological clinic of psychiatrist Henricus Rümke (1893–1967) in Utrecht. He had discussions with several of Rümke's colleagues on the diagnosis and treatment of children with behavioral problems. From Utrecht, Bowlby went to Nijmegen to visit the *Paedologisch Instituut St. Joseph*, led by psychiatrist A. P. J. Meyknecht, who was a strong advocate of the ideas of Alfred Adler (1870–1937) and emphasized the influence of the environment on the development of children. In the 1930s, the institute was one of the first to diagnose “autism” in young children (Van Drenth, 2018).

On Saturday, January 28, Bowlby visited psychologist Jacob Koekebakker (1907–1981) in Leiden. Koekebakker, together with Mulock Houwer, had been a driving force behind the establishment of child protection services in The Netherlands after the war. He was also doing some systematic research on juvenile delinquency, which Bowlby—given his own studies on juvenile thieves—found of interest, although it did not bring him any new insights.

In one of his letters, Bowlby sighed that because everybody in the Netherlands was inundated with case material and no one seemed to have time, or even the inclination, to do any systematic research, and he doubted if he would get much research findings “relevant to the topic in hand.” However, the theoretical position of the Dutch experts seems to have been much more in line with his own eclectic ideas than the more orthodox stance of the colleagues in Paris. Bowlby found that in Dutch child psychiatry people were doing excellent work and were generally working along the same lines as people at the Tavistock Clinic. Although some Dutch believed Bowlby was “not Freudian enough” (Van der Horst, 2014), the general idea in Holland was that mental ill‐health in children was the outcome of an inadequate rearing environment and that Child Guidance work should focus on the prevention of behavioral problems by helping the parents. This clear focus on the environment of children was one of the basic notions of the Mental Hygiene Movement that influenced child guidance work in England as well. But even though Bowlby agreed with the Dutch psychiatrists and social workers in child guidance and made use of Querido's and Mulock Houwer's findings, people like Van Meurs, Van der Spek, Hart de Ruyter, Lekkerkerker, Rümke, Meyknecht, and Koekebakker were not mentioned by name in the WHO report.

In the Netherlands, too, Bowlby found time to relax. On the last day of his visit, Bowlby walked around the old town of The Hague and visited the *Mauritshuis*, “a pleasant small gallery with about a dozen delightful pictures—Hobbema, Vermeer, Cuyp, Steen, Wouwerman^7^ and some early pictures as well.” He concluded that it had “really been rather an energetic week.” The next morning, he took a plane from Amsterdam to Stockholm.

### 2.4 Sweden: The importance of the social environment

On January 30, Bowlby arrived in Stockholm, his final destination on the European trip. Bowlby's informal host during the visit to Sweden was René de Monchy, a Dutch psychoanalyst, who lived and worked in Stockholm between 1943 and 1952. De Monchy picked him up at the airport and took him first to Hotel Plaza, located in the center of Stockholm. Then they continued to an evening gathering with a group of Swedish child psychiatrists and child psychoanalysts where Bowlby was formally welcomed to Sweden. Though Bowlby could not understand a word of the speech, the meeting itself was a good opportunity to meet people. Bowlby was happy about the reception he got and had the impression that everything in Sweden was well‐planned so that he did not have to waste time. During the meeting, Bowlby first heard about the discord between different groups within Swedish psychiatry and he noted that “the psychoanalytic point of view is having difficulty here against strongly entrenched ‘hereditary' views.” In this connection, he hoped that the psychoanalysts would enjoy “the cachet they g[o]t from a W.H.O. consultant being an analyst!”

In the next 2 days, Bowlby visited a number of different Child Guidance Clinics in Stockholm, whereas also having some time to relax, such as taking a walk around a snowy Stockholm, seeing the modern town hall and the Royal Palace. As for Child Guidance work, Bowlby was particularly impressed by the privately run *Erica Foundation* see (Bergenheim, 2013; Zetterqvist Nelson, 2011), but he also appreciated the Child Guidance Clinic attached to the *Pediatric Clinic* at *Norrtull Hospital* as well as the *Stockholm Township Educational Child Guidance Clinic* (Jönsson, 1997). Bowlby's first days in Stockholm included several dinner parties: for instance one organized by child psychiatrist Torsten Ramer, who at the time was a vice‐chair of the *International Association of Child Psychiatry*. In general, Bowlby was enthusiastic about the clinical child guidance activities going on in Stockholm and in a letter to Noel Hunnybun he commented that their methods of work were very comparable to Tavistock methods.

On February 2, Bowlby visited Skå, a recently started institution located outside Stockholm, under the leadership of Swedish child psychiatrist Gustav Jonsson. Bowlby spent the entire day at Skå, where he learned about the treatment activities, the organization, as well as the political conflict surrounding the institution. Skå had become famous because of its child‐oriented treatment ideology, which drew on psychoanalytical thinking, including encouraging children to express their emotions, even aggression, and sexuality. These ideas had led to outbursts in the media, which in turn resulted in political turmoil concerning the legitimacy of Skå. In a letter to Noel Hunnybun, Bowlby described Skå as an analytically oriented residential school, where severely disturbed children lived in small cottages and received psychotherapy. Bowlby continued that “in principle, it is an admirable set‐up and exactly what is wanted in England.” He concluded that, “unfortunately, the Society of the thing has been badly handled and this school, Skå (pronounced Sko) has become the center of bitter political controversy.”

From Bowlby's point of view, the political conflict was of less interest; the important thing about Skå was the psychoanalytical treatment approach. Bowlby was intrigued by the idea of allowing children to regress, something he specifically took notes on and mentioned twice in his WHO report. While being placed at Skå, all children were allowed to drink from a “babies bottle, which they enjoy, even a boy of 8–10 y[ea]rs.” Bowlby's fascination with the phenomenon is understandable but we have no evidence that he ever used it as a therapeutic intervention in his own psychiatric work. Bowlby also took special notice of the fact that Skå aimed to create small homelike institutions with a “house mother” and a “house father,” even though in the published report he emphasized the role of the “house mother” while downplaying the “house father.”

Later the same day, after returning to Stockholm, Bowlby had a meeting with Torsten Thysell, a child psychiatrist with a neuropsychiatric background, head of the child psychiatric clinic in Värmland county, located northwest of Stockholm. In a letter to Hunnybun, Bowlby gave an account of their meeting and he mocked Thysell's theories about how brain infections and other bodily conditions in children could cause neurotic behavior. However, Bowlby did show an interest in Thysell's (1948) research on children in institutions, as he took the raw data from Thysell's study and included a reference to his article in the WHO report. Thysell's study aimed to examine the relationship between institutional children's social background and upbringing and their later display of neurological problems. Bowlby was not interested in the latter issue and only used data on the causes of the children being deprived of a normal homelife. Bowlby's (1952), pp. 170‐172) critique of Thysell's report concerned the use of concepts such as “broken home,” which according to Bowlby was unclear; it did not specify when the separation between the mother and child had taken place, something Bowlby laid special emphasis on.

On February 5, Bowlby visited another institution, *Nybodahemmet* or *Nyboda*, which was located just outside Stockholm. At *Nyboda* he was shown around by child psychiatrist Sven Ahnsjö, a member of the Executive Board of WHO, who was later appointed the first Swedish professor in child psychiatry. Bowlby described *Nyboda* in the WHO report as a negative example of institutional care (Bowlby, 1952, p. 109). Though the majority of the children placed at *Nyboda* stayed there for a very short period, they were placed together with children who were there for a longer period, which in Bowlby's view was problematic. Bowlby rejected institutional care in general, but if absolutely necessary, it should be a brief stay in a small, home‐like institution. Sven Ahnsjö discussed institutional care with Bowlby, but the main socio‐political issue in Sweden at the time concerned another dilemma: should the responsible authorities focus on supporting the problematic family to take care of their own children or should they intervene and take the child away from the family? For Bowlby, this was not an issue. He argued in favor of the family, or to be more specific, in favor of maternity care, and clearly opposed all kinds of institutional care.

Bowlby spent the next day in Uppsala, approximately 60 km north of Stockholm, where Sweden's oldest university is located. Bowlby had an early meeting with Anna‐Stina Annell, a child psychiatrist with a neuropsychiatric orientation, at the child psychiatric department of the *Uppsala Hospital*. Bowlby's notes from this visit were very sparse and he seemed to take no interest in Annell's clinical approach to children with psychiatric problems. However, the next meeting with Gunnar Dahlgren seems to have raised Bowlby's interest. Dahlgren was the head of the state‐run *Rasbiologiska Institutet* in Uppsala. This institution, despite its ominous name, which suggests racist research, had at that time become the main center for advanced research in medical genetics. Dahlgren, who was openly supporting the Social Democratic government, was an important agent in the scientific development of advanced statistics.

Looking at the WHO report, it is evident that Bowlby was interested in the research carried out under the supervision of Dahlgren, such as Ahnsjö's (1941) study on social‐psychiatric aspects of delinquency in girls, and in Otterström's (1946) study of delinquent youth with adverse family background. These studies were included in the report, because their methodological design corresponded with Bowlby's call for robust scientific empirical work. However, a careful reading of the way in which Bowlby referred to the work of Otterström, Ahnsjö, and Thysell, demonstrates that he nevertheless rejected their work on other grounds. Although they lived up to Bowlby's standard of scientific rigor and statistical methods, neither Ahnsöj's nor Otterström's focus on the relationship between social factors, such as housing or education, and children's mental problems, or Thysell's focus on neuropsychiatric factors, appealed to Bowlby. He was also critical of their lack of strict definitions concerning mother–child separation.

Nevertheless, Bowlby was satisfied with his visit to Sweden and he wrote to his wife that he considered his visit to Stockholm a great success. Bowlby was “pretty sure [he'd] given great encouragement to psychoanalysis and dynamic psychiatry, which is a rather hostile world needs all the help they can get.” However, this was not the goal of his visit and on closer inspection, it seems that the net result of the visit to Sweden for the WHO report was limited. The reason is that there seems to have been a mismatch between Bowlby and the Swedish child psychiatric experts. At first sight, Bowlby seemed to agree with many Swedish child psychiatrists on the nonhereditary, social causes of children's mental health problems, but on closer scrutiny he appeared to reject their broad approach, which included such factors as poor housing, unemployment, and parental alcohol abuse, and focused exclusively on mother‐child bonding. Such an exclusive focus raised eyebrows in the social democratic Sweden of the 1950s, which traditionally saw it as its task to improve the lot of the less privileged in society. This mismatch between Bowlby and Swedish experts about the importance and extent of the social environment for children's mental health explains why Bowlby paid only scarce attention to Swedish views in his WHO report and why this report and his later ideas about the importance of mother–child attachment initially met with little enthusiasm in Sweden (Zetterqvist Nelson, 2009; Zetterqvist Nelson et al., 2017).

### 2.5 Back to Geneva and onward to the USA

After leaving Stockholm, Bowlby returned to the WHO headquarters in Geneva. During his meetings with experts throughout Europe, he had had no time for any consecutive thought. As a result, he felt “rather like a sponge having collected all sorts of ideas from all sorts of people,” and was “quite unable to sort it all out in [his] own mind.” Back in Geneva, he started writing up a preliminary report on the psychiatry and psychotherapy in the three countries he had visited. Although this was not what he had initially planned, to Bowlby it seemed “stupid” not to make a detailed record of everything he had read and heard, which would serve as a basis for future analysis.

At the WHO headquarters, Bowlby again had the opportunity to discuss his preliminary results with Hargreaves, which he did on several occasions. For instance, he dined with Ronald and talked about the future of the Tavistock, for which he had some new ideas. During his layover at the WHO Bowlby also planned his 6‐week trip to the USA, a trip that would follow the visit to Europe. He set up meetings with leading researchers working across the Atlantic, such as Harry Bakwin, René Spitz see (Van der Horst, Van Rosmalen, & Van der Veer, 2019), Henry Murray, Edward Tolman, Lauretta Bender, and Bruno Bettelheim (see Van Rosmalen, Van der Veer, & Van der Horst, in press).

## 3 CONCLUSIONS

In this contribution, on the basis of a detailed reconstruction of Bowlby's encounters with clinicians and researchers in the field of child guidance and child psychiatry in several European countries, we showed how Bowlby gathered evidence for his monograph *Maternal Care and Mental Health* (Bowlby, 1952). It became evident that Bowlby was not a passive recipient of ideas he heard from others, but selectively used the relevant sources and followed his own special interest in mother–child separation. During the writing of the report, Bowlby gradually moved away from the original, broader focus of the assignment on mental health aspects of homeless children who “need care in foster homes, institutions or other types of group care” to the much narrower focus on physical maternal deprivation and its consequences for children's mental health.

The result was a report that discussed the issue of deprivation in two parts: the first part dealing with the adverse effects of maternal deprivation, the second with the prevention of maternal deprivation. Bowlby's main conclusion was “that the prolonged deprivation of the young child of maternal care may have grave and far‐reaching effects on his character and so on the whole of his future life,” though he admitted that there were “still far too few systematic studies and statistical comparisons in which proper control groups ha[d] been used” (p. 46). As we have seen, a case in point was Bowlby's own use of Mulock Houwer's very preliminary findings on foster placement. Based on studies and observations by several others, Bowlby also noted “that deprivation occurring in the second half of the first year of life is… of great significance,” but that “comparative success of many babies adopted between 6 and 9 months who have spent their first half‐year in conditions of deprivation makes it virtually certain that, for many babies at least, provided they receive good mothering in time, the effects of early damage can be greatly reduced” (pp. 48‐49). Here, the findings of Roudinesco's team in Paris on the development of children who were placed in foster care proved very valuable.

That Bowlby selectively used the information and arrived in various countries with well‐established views became evident at various points. As can be concluded from his notes on his Swedish visit, for instance, he absorbed the information that he could assimilate into his own rather stable frame of mind concerning the importance of early mother–child bond and disregarded the involvement of a housefather in Skå. He also ignored the broader view of the social origin of children's mental health problems typical of postwar Sweden. In his final report, Bowlby also more or less disregarded the orthodox psychoanalytic views predominant in French psychiatry, for instance, concerning the child's inner world and fantasy life—something that anticipated his later move away from psychoanalysis (Van der Horst, 2011). His focus in *Maternal care and mental health* was on the influence of the environment on children's mental development, with an almost exclusive emphasis on the mother–child bond. The fact that Bowlby, as an assumed expert in the field of juvenile delinquency, refused Hargreaves' first offer to study delinquency and waited for the opportunity to study homelessness and separation confirms his huge interest in the theme. Bowlby had conceived the idea of the crucial role of the mother–child relationship very early in life (Van der Horst & Van der Veer, 2010) and admitted to having “a rather one‐track, one‐problem mind” (Tanner & Inhelder, 1971, p. 27). As a consequence, his later attachment theory met with skepticism in some of the recipient countries, in a way that reflects different views on the significance of mother–child relation, as well as nation‐specific policies for child psychiatric care. As we have seen, the WHO report itself also elicited methodological criticism and was followed by a new report 10 years later.

However, in the long run, Bowlby profited from his appointment with the WHO in many ways. First of all, he was able to get research findings from the people he met in Switzerland, Holland, France, and Sweden to further develop his ideas on separation and deprivation (cf. Polat, 2017). Second, he was able to see what clinical work was being done on the continent and what interventions were useful for the work done at “his” Tavistock Clinic. Finally, and not the least important, through his WHO contacts he was able to secure funding for his further research on mother‐child separation. In sum, the WHO appointment contributed considerably to the spread of Bowlby's ideas about the origin of children's mental health problems and helped him to adduce evidence for what would later develop into attachment theory—also as a result of Bowlby handing out reprints of his own work at the first opportunity. On the other hand, these ideas themselves seem to have changed very little over the years. On the basis of the detailed reconstruction of Bowlby's meetings and discussions with foreign experts in various European countries and their reflection in the WHO report one gets the idea that Bowlby was primarily seeking confirmation of his views.

To recapitulate, in Paris Bowlby spoke with orthodox psychoanalysts whose work he opted to neglect in his final report. In Amsterdam he met with people who shared his views but couldn't provide him with scientific research data. In Stockholm, he was confronted with a much broader social view on the origin of and remedy for homeless children, which he chose to ignore. Moreover, throughout this paper we have seen that Bowlby was somewhat skeptical about the possible results of his research trip and that he feared he might end up wasting his time with pretty stupid' colleagues. The question, then, becomes whether Bowlby's trip was an open‐minded search for new scientific data as to homeless children or whether he looked for confirmation of his firm conviction that the child's separation from his mother was the cause of all problems. Having read all Bowlby's notes and letters about the meetings with experts that we were able to reconstruct it seems that for him the WHO trip was first and foremost an excellent opportunity to find research data that confirmed his views and to spread his ideas. And in this respect, the trip and the report were a complete success.

## ENDNOTES

1 Bowlby kept track of reviews of his WHO monograph and compiled a scrapbook with more than 30 press cuttings (PP/BOW/A.4/1; e.g., Clarke, 1952; Editorial, 1951a; 1951b; 1951c; Editorial, 1952; Simon, 1951).

2 Northolt Airport is a small military airport on the western edge of London that has now largely been superseded by Heathrow.

3 In this reconstruction we make use of Bowlby's notebooks (AMWL: PP/BOW/D.4/8), which contain careful notes about the people he met, their studies of potential interest, and short sections on their personal opinions and impressions. Additionally, we use the more detailed reports, dictated by Bowlby and subsequently sent to Noel Hunnybun, a senior psychiatric social worker at the Tavistock Clinic in London (AMWL: PP/BOW/B.1/11). Finally, we draw on Bowlby's correspondence with his wife Ursula Bowlby, who showed a strong involvement in his work (cf. Kahr, 2016).

4 A British‐built bakelite dictating machine launched in 1948. Words were magnetically recorded on paper disks, which could be folded and sent home in an envelope. It is unknown whether some of these disks have been preserved.

5 Philippe Kocher would remain active in the Swiss psychoanalytic movement until the 1990s and published regularly in the psychoanalytic press, mainly as a translator, making available in French the English psychoanalytic works of people like Joseph Sandler, Wayne Myers, Georges Abraham, Janice de Saussure, and Léon Wurmser.

6 Kees van Dongen (1877–1968) was a Dutch‐French “Fauvist” painter, who lived in Paris for most of his career. It is unclear whether the Roudinesco's had paintings of either Jean Dufy (1888–1964) or Raoul Dufy (1877–1953) in their appartment, although the work of the latter was more similar to that of Van Dongen.

7 Meindert Hobbema (1638–1709), Jan Vermeer (1632–1675), Aelbert Cuyp (1620–1691), Jan Steen (1626–1679), and Philips Wouwerman (1619–1668) were all painters during the Dutch Golden Age.
